# Flavonoid Profiles and Antioxidant Potential of *Monochoria angustifolia* (G. X. Wang) Boonkerd & Tungmunnithum, a New Species from the Genus *Monochoria* C. Presl

**DOI:** 10.3390/antiox11050952

**Published:** 2022-05-12

**Authors:** Duangjai Tungmunnithum, Samantha Drouet, Laurine Garros, Jose Manuel Lorenzo, Christophe Hano

**Affiliations:** 1 Department of Pharmaceutical Botany, Faculty of Pharmacy, Mahidol University, Bangkok 10400, Thailand; 2 Laboratoire de Biologie des Ligneux et des Grandes Cultures, INRAE USC1328, Campus Eure et Loir, Orleans University, 28000 Chartres, France; samantha.drouet@univ-orleans.fr (S.D.); laurine.garros@univ-orleans.fr (L.G.); 3 Le Studium Institue for Advanced Studies, 1 Rue Dupanloup, 45000 Orléans, France; 4 Centro Tecnológico de la Carne de Galicia, Adva. Galicia no. 4, Parque Tecnológico de Galicia, San Cibrao das Viñas, 32900 Ourense, Spain; jmlorenzo@ceteca.net; 5 Área de Tecnología de los Alimentos, Facultad de Ciencias de Ourense, Universidad de Vigo, 32004 Ourense, Spain

**Keywords:** *Monochoria angustifolia*, *Monochoria hastata*, flavonoid, antioxidant mechanism, natural populations, phytochemical profile, traditional herbal medicine, phytopharmaceutical application

## Abstract

Plants of the genus *Monochoria* have long been utilized in food, cosmetics, and traditional herbal treatments. Thailand has the highest species diversity of this genus and a new member, *Monochoria angustifolia* (G. X. Wang) Boonkerd & Tungmunnithum has been recently described. This plant is called “Siam Violet Pearl” as a common name or “Khimuk Si Muang Haeng Siam” as its vernacular name with the same meaning in the Thai language. Despite their importance, little research on *Monochoria* species has been conducted. This study, thus, provides the results to fill in this gap by: (i) determining flavonoid phytochemical profiles of 25 natural populations of *M. angustifolia* covering the whole floristic regions in Thailand, and (ii) determining antioxidant activity using various antioxidant assays to investigate probable mechanisms. The results revealed that *M. angustifolia* presents a higher flavonoid content than the outgroup, *M. hastata*. Our results also revealed that flavonoids might be used to investigate *Monochoria* evolutionary connections and for botanical authentication. The various antioxidant assays revealed that *M. angustifolia* extracts preferentially act through a hydrogen atom transfer antioxidant mechanism. Pearson correlation analysis indicated significant correlations, emphasizing that the antioxidant capacity is most probably due to the complex action of several phytochemicals rather than that of a single molecule. Together, these results showed that this new species provide an attractive alternative starting material with phytochemical variety and antioxidant potential for the phytopharmaceutical industry.

## 1. Introduction

*Monochoria angustifolia* (G. X. Wang) Boonkerd & Tungmunnithum, is the newest member of the genus *Monochoria* C. Presl which belongs to the family Pontederiaceae. There are eight species of *Monochoria* worldwide [1,2,3], and Thailand is the richest species diversity area of the genus *Monochoria*. There are four species reported in Thailand such as *M. elata*, *M. hastata*, *M. vaginalis* and *M. angustifolia* [1,2,3,4]. Currently, *M. angustifolia* is the new species that was described by Tungmunnithum and her research teams in 2020 based on both morphological and molecular (phylogenetic analysis) evidence [1].

*M. angustifolia* is an aquatic plant with beautiful blooming violet-pearl perianth and it is a new species from Thailand [1]. Therefore, this plant is called “Siam Violet Pearl” as a common name or “Khimuk Si Muang Haeng Siam” as a vernacular name with the same meaning in the Thai language. The natural habitat of the *M. angustifolia* plant is standing bodies of water in tropical regions. This new species is an annual herb that is distributed in several floristic regions in Thailand, and has never been reported in other countries (World Checklist of Selected Plant Families [1,2,3,4,5]. It is possible that environmental factors play an important role as the limiting factor for the distribution of *M. angustifolia* plants similar to other endemic species that are found only in Thailand, for example *Hoya siamica* Craib [6]. *M. angustifolia* populations have a narrow range of distribution compared with the other species in the same genus, but each population consists of a large number of individual plants. This plant species is native and well distributed throughout Thailand, easy to grow and can be propagated both sexually by seeds and asexually by budding. These characteristics are particularly suitable for research and development of raw plant materials for the phytopharmaceutical and cosmetic industries. Besides, it is also helpful for local people to grow and cultivate the potential population of *M. angustifolia*, so as to provide quality plant materials for the industrial sector. Furthermore, the *Monochoria* plant group has long been used as food (vegetable and/or cooking ingredient), skin care (leaves and flowers extracts) and traditional medicine (leaves and/or the aerial part) in Thailand, Japan, India and other countries in Asia since ancient times. Local people have used its leaves for the treatment of asthma and to relieve toothache. Its roots are also used to cure stomach and liver problems [4,7]. 

Flavonoids and other related phenolic substances are naturally occurring chemicals in plants [8,9,10]. Their antioxidant activity has been extensively studied, and it is widely assumed that it is connected to their quantity and/or chemical structures, such as the location of hydroxyl groups. However, the majority of information on antioxidant activity is generally based on a small number of plant species or cultivars [11]. Furthermore, antioxidant activity is usually determined using a restricted number of assays. Because of the complex structures of plant-derived compounds, as well as the fact that antioxidant activity is mostly dictated by the reaction mechanism involved, this biological activity cannot be assessed by using a single assay [12,13]. Furthermore, environmental and agricultural cultivation conditions such as location, soil conditions, and/or climate have been reported to have a significant impact on the phenolic compound accumulation as well as the antioxidant potential of a plant extract [11,14,15]. Thailand is located in “Indo-Burma” a biodiversity hotspot that is recognized as the world′s eighth most bio-diverse area. The country has one of the highest levels of biodiversity per unit area in the world. Thailand, which has a variety of forest types and aquatic environments, supports up to 10,000 plant species and accounts for around 10% of all living organisms on the earth [16]. So far, no study of this new species has addressed the diversity in flavonoids, other associated phenolic compounds, or antioxidant activities (measured using different assays capable of accounting for this biological activity). 

The objective of this study is to complete this knowledge, with natural *M. angustifolia* and the outgroup from the same genus, *M. hastata* populations originating from different floristic regions in Thailand, by (1) determining total phenolic content and total flavonoid content (including HPLC analysis of their flavonoid profiles), and (2) investigating the antioxidant potential using five in vitro antioxidant assays covering different antioxidant mechanisms as well as one yeast cell-based cellular antioxidant approach. 

## 2. Materials and Methods

### 2.1. Chemicals and Reagents

For extraction HPLC analysis and biological assays, all solvents and reagents were analytical grade or of the greatest purity possible (Thermo Fischer Scientific, Illkirch, France). A Milli-Q water-purification system was used to purify ultrapure deionized water (Merck Millipore Fontenay sous Bois, Paris, France). Prior to use, all HPLC solutions were filtered using 0.45 m nylon syringe membranes. Flavonoid standards (apigenin-7-*O*-rutinoside; luteolin-7-*O*-glucoside; apigenin-7-*O*-glucoside (i.e., apigetrin); luteolin; apigenin) were provided by Extrasynthese (Genay, France). These analytical standards were provided with *w*/*w* absolute assay (with purity of at least 97%), to be used for quantitative titrations.

### 2.2. Plant Material

The living plant specimens were searched for and collected from natural habitats covering the entire floristic regions in Thailand such as (1) Northern (2) North-eastern (3) Eastern (4) South-western (5) Central (6) South-eastern and (7) Peninsular. The collected populations were named according to the collected provinces (Table 1). The collected specimens were identified by using the taxonomic and the species description from existing flora and previous published works [1,4,7,17,18,19], and then compared with the herbarium specimens kept in the Forest Herbarium (BKF), Kyoto University, Japan (KYO), the Prof. Kasin Suvatabandhu Herbarium, Chulalongkorn University, Bangkok, Thailand (BCU) and the Plant Varieties Protection Office, Bangkok, Thailand (BK). The herbarium abbreviations are used according to Thiers [20]. The leaves of each plant material sample were air-dried, and then prepared using the recommendations of the World Health Organization [21]. The total 25 populations of *M. angustifolia* (15 samples/population) collected from every floristic region throughout the country as well as the outgroup (*M. hastata*) shown in Table 1 were included in this study. 

### 2.3. Extraction

The dried leaf (100 mg) material [11,22] was placed with 1 mL of 90% (*v*/*v*) aqEtOH into a 5 mL quartz tube which was capped with a vapor condenser, and extracted by ultrasound using a USC1200 TH ultrasonic bath (Prolabo, Fontenay-sous-Bois, France). The extraction conditions used were US frequency 30 kHz, a duration 45 min, and temperature 45 °C. The obtained extracts were cooled at 25 °C, centrifuged at 5000× *g* for 15 min (Heraeus Biofuge Stratos, Thermo Scientific, Illkirch, France), and the supernatant filtered through 0.45 m nylon syringe membrane (Merck Millipore, Saint-Quentin Fallavier, France). The previously described DAX-8 macroporous resin (Merck Millipore, Saint-Quentin Fallavier, France) purification technique was then used for flavonoid enrichment [22].

### 2.4. Determination of Total Phenolic Content (TPC)

Determination of TPC was done by the Folin–Ciocalteu method adapted for a microplate reader as previously reported [11] with absorbance determined at 650 nm (BioTek ELX800 Absorbance Microplate Reader, BioTek Instruments, Colmar, France). The TPC was expressed in gallic acid equivalents per 100 g dry weight (mg GAE/100 g DW) using a standard curve of gallic acid (linear range: 0–40 g/mL; R^2^ = 0.998) (Merck, Saint-Quentin Fallavier, France).

### 2.5. Determination of Total Flavonoid Content (TFC)

The TFC was determined by an aluminum trichloride (AlCl_3_) colorimetric technique adapted for a microplate reader (Multiskan GO, Thermo Fischer Scientific, Illkirch, France) as previously described [11], with absorbance read at 415 nm. The TFC was then expressed as mg of quercetin equivalents per 100 g dry weight (mg QE/100 g DW) using a standard curve of quercetin (Merck, Saint-Quentin Fallavier, France) (linear range: 0–40 g/mL; R^2^ = 0.999).

### 2.6. High-Performance Liquid Chromatography (HPLC) Analysis

The HPLC system used was a Varian HPLC system controlled by Galaxie software (Varian v1.9.3.2, Varian, Le Plessis-Robinson, France). The system is composed of an autosampler, Varian Prostar 230 pump and Varian Prostar 335 photodiode array detector (PDA). The separation was carried out at 40 °C with a Purospher RP-18 column (250 × 4.0 mm internal diameter; 5 µm) (Merck Chemicals, Molsheim, France) using a linear gradient of methanol (solvent A) and HPLC grade water acidified with 0.05% formic acid (solvent B) from a 5:95 (*v*/*v*) mixture of solvents A and B to a 100:0 (*v*/*v*) mixture at a flow rate of 0.8 mL/min for 60 min. Injection volume was 3 µL. The highest back pressure was 110 bar. The detection for quantification was performed at 320 nm. Quantification was accomplished using commercial authentic flavonoid standards (Extrasynthese, Genay, France).

Validation was performed as described previously by Tungmunnithum et al. [23] and according to the Association of Analytical Communities (AOAC) standards, so as to ensure the accuracy and the reproducibility of quantification [24].

### 2.7. In Vitro Cell Free Antioxidant Assays

The antioxidant potential of extracts was assessed by using five different in vitro cell free antioxidant assays: ABTS (2,2-azinobis(3-ethylbenzthiazoline-6-sulphonic acid), DPPH (2,2-diphenyl-1-picrylhydrazyl), ORAC (oxygen radical absorbance capacity assay), FRAP (ferric reducing antioxidant power) and CUPRAC (cupric reducing antioxidant capacity), as described previously [12,13]. 

FRAP assay: 10 µL of extract was added to 190 µL of FRAP reagent (10 mM TPTZ, 20 mM FeCl_3_.6 H_2_O, 300 mM acetate buffer pH 3.6, in a ratio of 1:1:10 (*v*/*v*/*v*)) in a microplate well, mixed and incubated for 15 min at 25 °C in the dark. 

DPPH assay: 20 μL of extract was added to 180 μL of DPPH reagent (0.1 mM final concentration in MeOH) in a microplate well, mixed and incubated for 15 min at 25 °C in the dark.

CUPRAC assay: 10 μL of extract was added to 190 µL of CUPRAC solution (10 mM Cu(II), 7.5 mM neocuproine, and 1 M acetate buffer pH 7 mixed in ratio 1:1:1 (*v*/*v*/*v*)) in a microplate well, mixed and incubated for 15 min at 25 °C in the dark.

For ABTS assay: 10 μL of extract was added to 190 µL of ABTS solution (7 mM ABTS, 2.45 mM potassium persulphate) in a microplate well, mixed and incubated for 15 min at 25 °C in the dark.

After incubation, absorbances at 590 nm (FRAP), 450 nm (CUPRAC), 515 nm (DPPH) and 734 nm (ABTS) were determined using a microplate reader (BioTek ELX800 Absorbance Microplate Reader, BioTek Instruments, Colmar, France). The antioxidant activity was then expressed in µmoles of Trolox C equivalent antioxidant capacity (µM TEAC) with a standard curve (R^2^ = 0.998–0.999, 0–500 μM Trolox C) for each assay.

For ORAC (oxygen radical absorbance capacity assay), 10 µL of extract was added to 190 µL of ORAC reagent (0.96 µM fluorescein in 75 mM phosphate buffer pH 7.4), mixed, and incubated at 37 °C for at least 30 min with shaking. Then, 20 µL of 119.4 mM 2,2′-azobis-amidinopropane (ABAP, Sigma Aldrich, Saint-Quentin Fallavier, France) was added and the fluorescence intensity was measured every 5 min for 2.5 h at 37 °C using a fluorescence spectrophotometer (Bio-Rad, Marnes-la-Coquette, France) set with excitation wavelength at 485 nm and emission wavelength at 535 nm. Antioxidant capacity was represented as Trolox C equivalent antioxidant capacity in triplicate assays (TAEC).

### 2.8. Cellular Antioxidant Assays

The protocol described in Nazir et al. [25], employing yeast cells, was used to assess cellular antioxidant activity. The yeast DBY746 strain (MAT leu2-3,112 his31 trp1-289 a ura3-52 GAI+) was aerobically cultivated in full Yeast extract Peptone Dextrose medium (YPD) with 2% (*w*/*v*) glucose (Sigma Aldrich, Saint-Quentin Fallavier, France) in 150 rpm of the orbital shaker at 30 °C. The extract was evaporated under a nitrogen flow, and dissolved in the DMSO solution, and added to yeast cells at a final concentration of 1 mg/mL, 6 h before oxidative stress induction. For untreated control yeast cells, the same amount of DMSO was utilized. The final DMSO dose administered to the yeast cells was around 1% (*v*/*v*). The UV-C irradiation; 106.5 J/m^2^ UV-C at 254 nm was used to generate oxidative stress using a Vilber VL-6.C filtered lamp (Thermo Fisher Scientific, Villebon-sur-Yvette, France). Then, the yeast cells were incubated overnight at 30 °C. Dihydrorhodamine-123 (DHR-123) fluorescent dye (Sigma-Aldrich, Saint-Quentin Fallavier, France) was used to assess the amount of reactive oxygen species (ROS), as well as the reactive nitrogen species (RNS) that were generated. Approximately 10^8^ yeast cells were then rinsed twice in phosphate buffered saline pH 7.4, before being resuspended in 0.4 M DHR-123 solution prepared in phosphate buffered saline pH 7.4 (1 X), and then incubated in the dark for 10 min at 30 °C. The intensity of fluorescence was determined using a VersaFluor fluorimeter (Biorad, Marnes-la-Coquette, France) (λem = 535 nm; λex = 505 nm) after twice washing with PBS (1 X, pH7.4).

### 2.9. Statistical Analysis

The statistical software packages: XLSTAT 2019 suite (Addinsoft, Paris, France) as well as PAST4.0 [26] were employed for statistical analysis. Data composed of at least 3 independent replicates were analyzed and then presented using the means and the standard deviations. The Student’s *t*-test statistic was calculated for comparative analysis. The significant differences at *p* < 0.05 and *p* < 0.01 as well as *p* < 0.001 were presented using *, **, and ***, respectively. The different letters indicate the statistical significance at *p* < 0.05.

## 3. Results and Discussion

### 3.1. Plant Collection and Taxonomic Description

After an intense search for living specimens in natural habitats, the 25 populations of *M. angustifolia* from different localities covered all floristic regions in Thailand and were collected along with the outgroup (six populations of *M. hastata*) from the same genus *Monochoria*. Samples investigated in the present study are listed in Table 1. 

**Figure 1:**
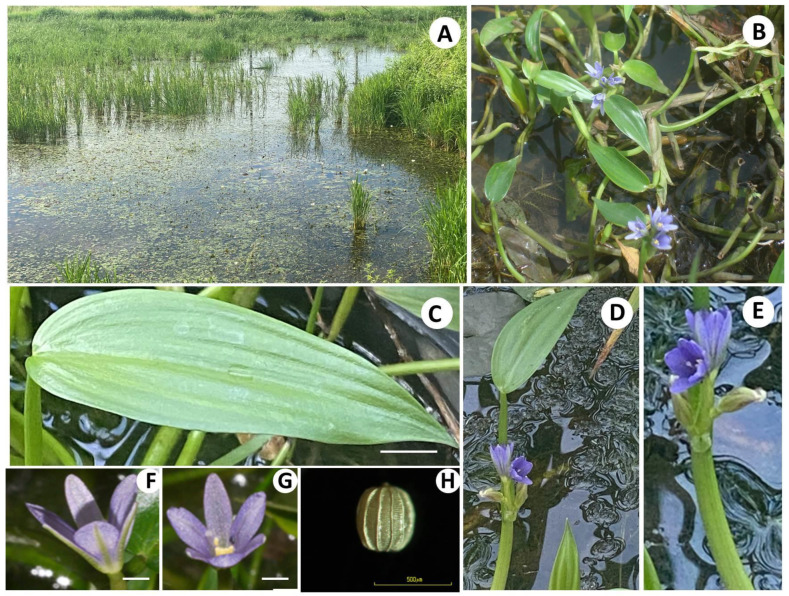
*M. angustifolia (***A**): Natural Habitat, (**B**): The whole plant with inflorescence, (**C**): Leaf, Bar scale = 0.5 cm; (**D**,**E**): Inflorescence, (**F**): Flower (side view) Bar scale = 0.3 cm; (**G**): Flower (top view) Bar scale = 0.3 cm; (**H**): Seed, Bar scale = 500 µm. All photos were taken in Thailand by D.T.

The 25 populations of *M. angustifolia* collected from all the seven floristic regions of the country showed a similar trend in morphological characters (Figure 1), and the taxonomic description is provided in the paragraph below. 

**Figure 2:**
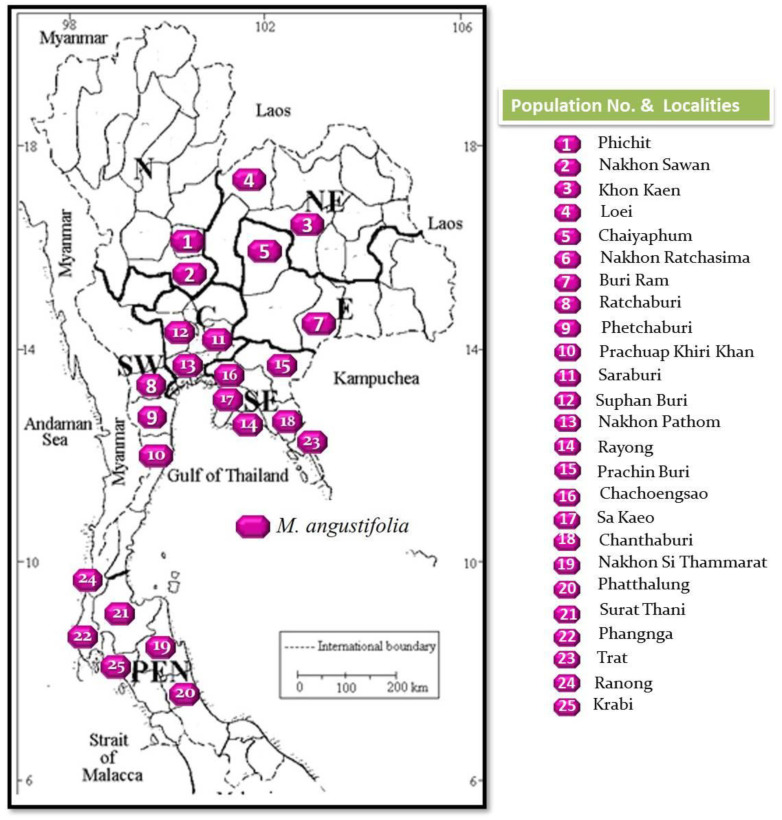
Distribution map of *M. angustifolia* populations throughout the floristic regions in Thailand. Numbers 1–25 indicate the number of populations.

According to the distribution map (Table 1 and Figure 2), *M. angustifolia* is mainly distributed throughout the Eastern, South-Eastern, Central, South-Western, and Peninsula, as well as some parts of the Northern and North-Eastern floristic regions which is wider than previously reported [1]. During our field study, at least five targeted provinces per floristic region where *M. angustifolia* was previously reported, or contained possible aquatic natural habitats, were selected and searched for specimens of this new species. However, *M. angustifolia* living plant specimens were found in only two localities (provinces) in the Northern (Phichit and Nakhon Sawan provinces) and North-Eastern (Khon Kaen and Loei provinces) floristic regions. The most abundant *M. angustifolia* can be found in the South-Eastern floristic regions where six populations were found.

**Taxonomic Description:** Annual aquatic herb, leaf simple, stipulate, green, glabrous; petiole rounded, erect or curved, with broad leaf sheath; leaf blade lanceolate, lanceolate-linear, or ovate-lanceolate, apex abruptly acuminate, base obtuse, 1.4–2.0 cm wide, 6.0−7.4 cm long, midrib groove on the adaxial surface, leaf blade and petiole forming right angle or acute to each other; Inflorescence racemose, 2−6 flowers; rachis 5.2−5.4 cm long; peduncle 2.8−3.0 cm long; spathe green, 2.6−2.8 cm long, terminal appendage approximately 0.3 cm long; floral leaf blade lanceolate, adaxial and abaxial surfaces smooth, midrib groove, apex acute, base obtuse, 1.7−1.8 cm wide, 6.3−6.7 cm long; floral leaf petiole green, 4.1−4.5 cm long, level of inflorescence tip higher than that of floral leaf and mature leaf; pedicel glabrous, 1.3−1.4 cm long; outer perianth 3, violet-pearl, glabrous, lanceolate, middle of abaxial green, 0.3 cm wide, 0.7−0.8 cm long; inner perianth 3, violet-pearl, ovate or elliptic, glabrous, apex obtuse, middle of abaxial green, 0.3 cm wide, 0.8−0.9 cm long; stamen 6; normal stamen 5, filament white unappendage, 0.7−0.8 cm long, anther basifixed, yellow, 0.4−0.5 cm long; largest stamen 1, filament dark purple, appendage, 0.2 cm long, anther basifixed, dark purple, 0.1 cm wide, 0.4−0.5 cm long; ovary superior, style bright purple, 0.4−0.5 cm long. Fruits capsule, glabrous. Seeds barrel, 373−429 µm long, numerous, longitudinal ridges of seed distinct, 7−10 veins. 

**Specimens examined:***M. angustifolia* Populations No. 1–25 

**Recent distribution:** Every floristic region

**Ecology:** Rice fields and clear aquatic habitats 

**Flowering Time:** Early April to early August.

### 3.2. Phytochemical Characterization

**Figure 3:**
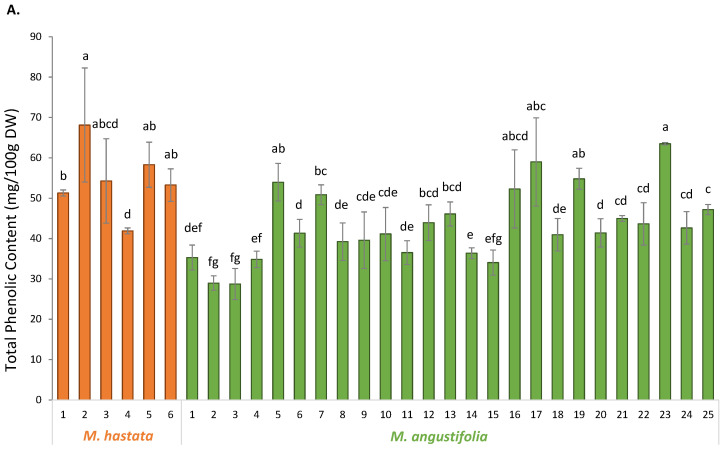

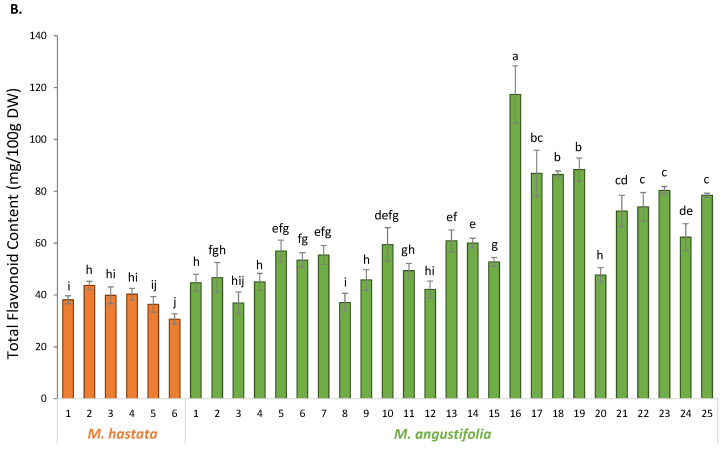
Total phenolic content (**A**) and total flavonoid contents (**B**) in different populations of two *Monochoria* species including the 6 populations of *M. hastata* and 25 populations of *M. angustifolia*) covering the entire floristic regions from Thailand. Different letters indicate significant differences at *p* < 0.05.

First, the TPC and TFC of the extracts from the different populations of the two *Monochoria* species (*M. angustifolia* and *M. hastata*) are presented in Figure 3 (TPC Figure 3A and TFC Figure 3B).

The TPC ranged from 28.78 ± 3.86 (Ma#3) to 63.49 ± 0.29 (Ma#23) mg per 100 g DW gallic acid equivalent in *M. angustifolia* and ranged from 41.91 ± 0.72 (Mh#4) to 68.12 ± 14.15 (Mh#2) mg per 100 g DW gallic acid equivalents in *M. hastata*. The TFC, ranged from 36.92 ± 4.26 (Ma#3) to 117.33 ± 11.18 (Ma#16) mg/100 g DW quercetin equivalents in *M. angustifolia* extracts, and from 30.68 ± 1.99 (Mh#6) to 43.68 ± 1.60 (Mh#2) mg/100 g DW quercetin equivalents in *M. hastata* extracts. These results revealed a significant degree of variability in both TPC and TFC for *M. angustifolia* as compared to *M. hastata*, with TFC playing a critical role in the phytochemical heterogeneity observed within the *M. angustifolia* populations. Noticeably, some populations of *M. angustifolia* appeared to be significantly richer in TFC, which might be of significant relevance given the well-known antioxidative and health-promoting effects of this class of phytochemicals [9,27]. In particular, population #16–19, #21–23 and #25 of *M. angustifolia* appeared of special interest because of their high TFC. Interestingly, this is the first report on the phytochemical examination of this new species. The TPC and TFC of the outgroup, *M. hastata* extracts, have been reported, and the published ranges of variations are in line with the present results [28,29]. It should be noted, however, that the present study is the first to evaluate TPC and TFC variabilities at the population level.

**Figure 4:**
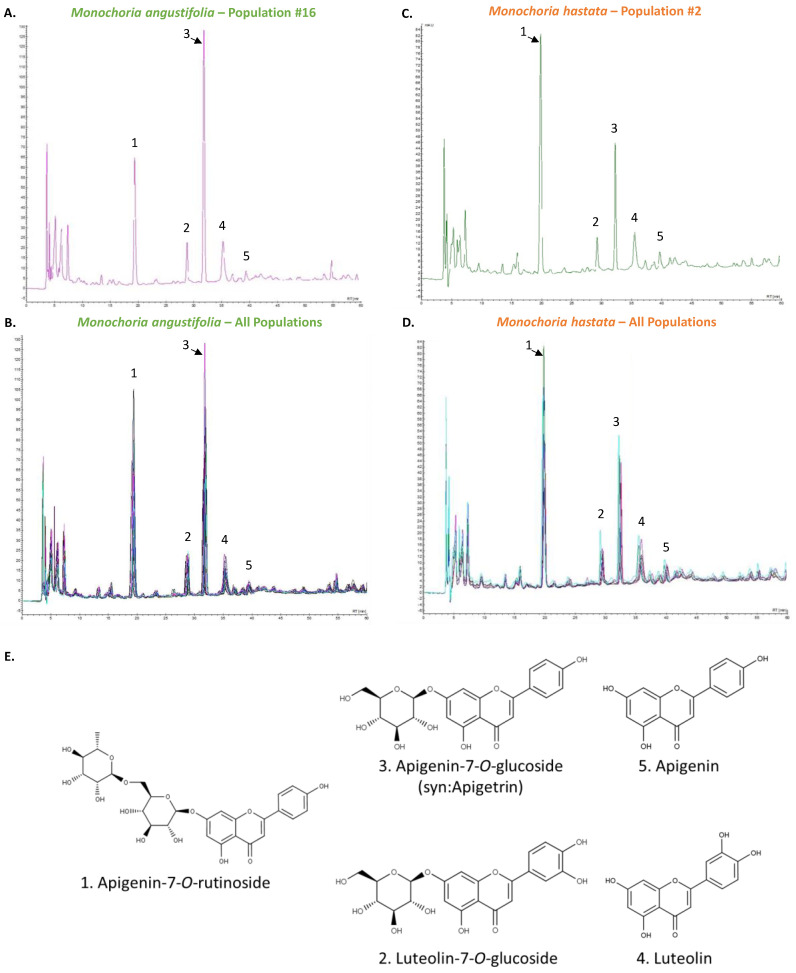
The HPLC chromatograms detected at 320 nm of (**A**) *M. angustifolia* population #16; (**B**) superimposed view of the *M. angustifolia* 25 populations; (**C**) *M. hastata* population #2; (**D**) superimposed view of the *M. hastata* 6 populations; (**E**) Structure of the main identified flavonoids: 1. apigenin-7-*O*-rutinoside; 2. luteolin-7-*O*-glucoside; 3. apigenin-7-*O*-glucoside (also known as apigetrin); 4. luteolin; 5. apigenin.

Our results indicated that TFC are important contributors to the observed variations, therefore HPLC coupled to PDA analysis was carried out in order to provide a thorough understanding of qualitative and quantitative changes (Figure 4). Compounds were identified using our flavonoid database by comparison with authentic commercial standards, based on retention times and λmax values.

The HPLC-PAD analyses allowed the identification of the five main flavonoids among the distinct *Monochoria* populations’ extracts: apigenin (compound 5) and two of its glycoside derivatives, apigenin-7-*O*-rutinoside (compound 1) and apigenin-7-*O*-glucoside (also known as apigetrin, compound 3), as well as luteolin (compound 4) and one of its glycoside derivatives, luteolin-7-*O*-glucoside (compound 2). 

Each of the compounds was well-separated with excellent repeatability, as can be seen in the relative standard deviations of the retention times with adequate peak symmetry and accurate resolution (Table 2). The LOD and LOQ values were determined using the response standard deviation and slope of the calibration curves and demonstrated that the proposed technique is adequately sensitive for measuring flavonoids from *M. angustifolia* leaf ethanolic extract (Table 2).

The method was then validated using the Association of Analytical Communities (AOAC) standards to ensure accuracy and reproducibility in quantification [24]. The validation results are summarized in Table 3, including the Horwitz ratio, accuracy, and intra- and inter-day precision. These results indicated that this analytical method is adequate to quantify the different flavonoid phytochemicals from *M. angustifolia* leaf extract.

The HPLC profiles (Figure 4) clearly illustrated that the accumulation profile differed qualitatively and quantitatively between the two *Monochoria* species. Indeed, while both species accumulated apigenin and luteolin primarily in their conjugated glycoside forms rather than their aglycone forms (compounds 4 and 5 on the HPLC chromatograms), the major conjugated form clearly differs between the two *Monochoria* species, with *M. hastata* samples accumulating apigenin-7-*O*-rutinoside (compound 1) as the main flavonoid, as opposed to *M. angustifolia* samples which accumulate apigenin-7-*O*-glucoside (compound 3) as the main flavonoid. Plants accumulate glycosidic forms to enhance their solubility and stability [30]. Therefore, this accumulation profile toward glycosidic forms makes sense. Interestingly, in some cases, glycosylation may improve flavonoid’s biological activities [27,30].

**Figure 5:**
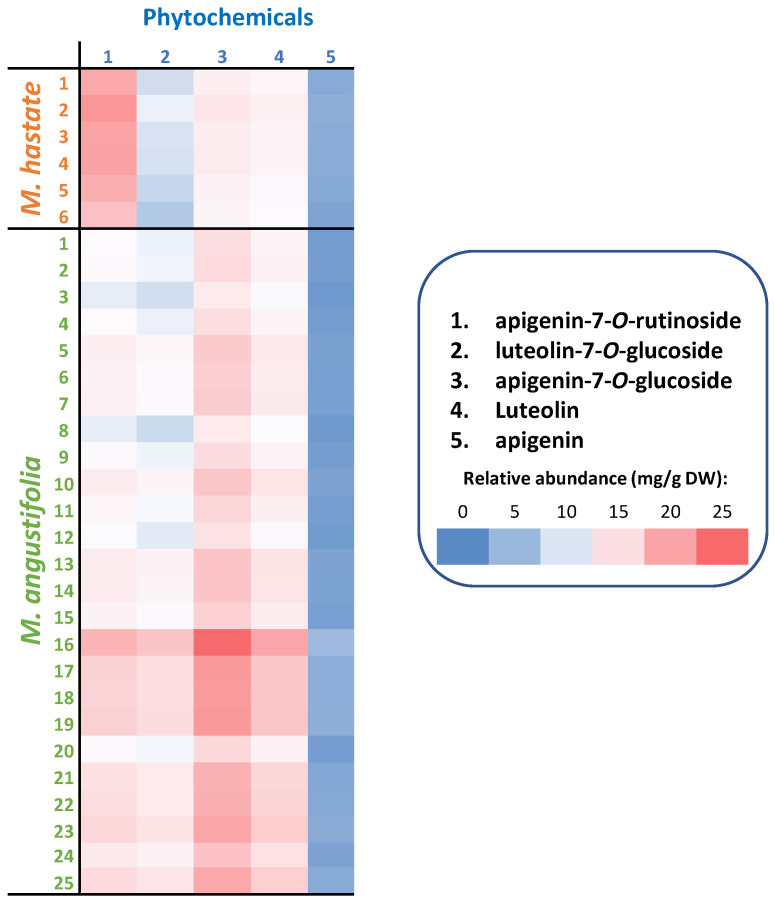
Absolute quantification of the main flavonoids in different populations of two *Monochoria* species (6 populations of *M. hastata* and 25 populations of *M. angustifolia*) covering the entire floristic regions from Thailand. 1. apigenin-7-*O*-rutinoside; 2. luteolin-7-*O*-glucoside; 3. apigenin-7-*O*-glucoside (also known as apigetrin); 4. luteolin; 5. apigenin. Means and standard deviations are provided in Table S1.

Absolute quantification of the five main flavonoids has been conducted (Figure 5, Table S1).

Individually, (1) apigenin-7-*O*-rutinoside ranged from 6.94 (Ma#3) to 22.04 (Ma#16) mg/100 g DW in *M. angustifolia* extracts, and from 19.78 (Mh#6) to 28.15 (Mh#2) mg/100 g DW in *M. hastata* extracts; (2) luteolin-7-*O*-glucoside ranged from 5.62 (Ma#8) to 18.91 (Ma#16) mg/100 g DW in *M. angustifolia* extracts, and from 3.43 (Mh#6) to 4.94 (Mh#2) mg/100 g DW in *M. hastata* extracts; (3) apigenin-7-*O*-glucoside ranged from 11.94 (Ma#3) to 36.94 (Ma#16) mg/100 g DW in *M. angustifolia* extracts, and from 9.63 (Mh#6) to 12.29 (Mh#2) mg/100 g DW in *M. hastata* extracts; (4) luteolin ranged from 7.86 (Ma#3) to 25.08 (Ma#16) mg/100 g DW in *M. angustifolia* extracts, and from 5.77 (Mh#6) to 7.28 (Mh#2) mg/100 g DW in M. hastata extracts; (5) apigenin ranged from 1.07 (Ma#3) to 3.41 (Ma#16) mg/100 g DW in *M. angustifolia* extracts, and from 1.83 (Mh#6) to 2.60 (Mh#2) mg/100 g DW in *M. hastata* extracts (Figure 5, Table S1). This is the first work on the HPLC analysis of the flavonoid content of *M. angustifolia* and, to the best of our knowledge, of *M. hastata* as well. However, glycoside derivatives of both apigenin and luteolin have been identified in a variety of species from the Pontederiaceae family [31,32,33], adding validity to our results. Future studies will be performed to identify other minor flavonoids using high resolution mass spectrometry. These results confirmed the different accumulation strategies observed with the TPC and TFC analyses for the two species, as well as at the population level in the case of *M. angustifolia*. The present results also revealed the special interest for possible applications of the new species (i.e., *M. angustifolia*), over *M. hastata* due to its higher flavonoid content. Many *Monochoria* species are invasive and considered weeds [34], however, this can be seen as a benefit in terms of obtaining a large and valuable biomass.

**Figure 6:**
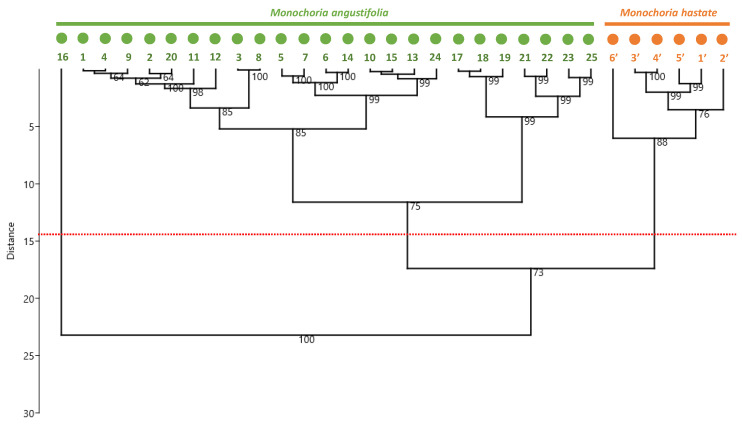
Hierarchical clustering analysis (HCA) of 25 different populations of *M. angustifolia* and 6 different populations of *M. hastata* on the basis of their phytochemical profiles. The percentages of replicate trees which associated samples cluster together in after bootstrap analysis (percentage of 5000 replicates) are shown next to the branches.

The observed variations may be the result of distinct genetic backgrounds, but they may also be the result of the effect of various ecological conditions. Environmental variables, for example climatic and geographic factors including growth conditions, in addition to genetic background, were demonstrated to be major influences on accumulation of phenolic compounds [14,15]. Hierarchical clustering analysis (HCA) was therefore used to discover probable groups among the heterogeneous samples from the various populations, in order to examine the structure of the populations (Figure 6).

Based on the phytochemical profiles, the HCA indicated that the clustering occurred predominantly at the genetic level, with a clear distinction between the two *Monochoria* species. In good agreement, flavonoids have been successfully employed to analyze evolutionary connections of various angiosperm families, and also for botanical authentication [35,36,37,38]. However, there is no discernible pattern to illustrate the importance of the environmental factors. Given the wide geographic distribution of different *M. angustifolia* populations through the diverse floristic regions of Thailand. We cannot rule out the possibility that environmental variables may explain at least some of the observed heterogeneity, but it appears that genetics is a major driver of the phytochemical diversity. Overall, the present results give the most comprehensive picture to date of the phytochemical, flavonoid-specific, broad variability found at different population levels of the two *Monochoria* species, including the newly described *M. angustifolia*. Flavonoids, through their antioxidant action, have been demonstrated to have a wide range of health-promoting properties [9]. As a result, we next investigated the impact of this wide flavonoid variability on the antioxidant activity of these extracts.

### 3.3. Antioxidant Activity

**Figure 7:**
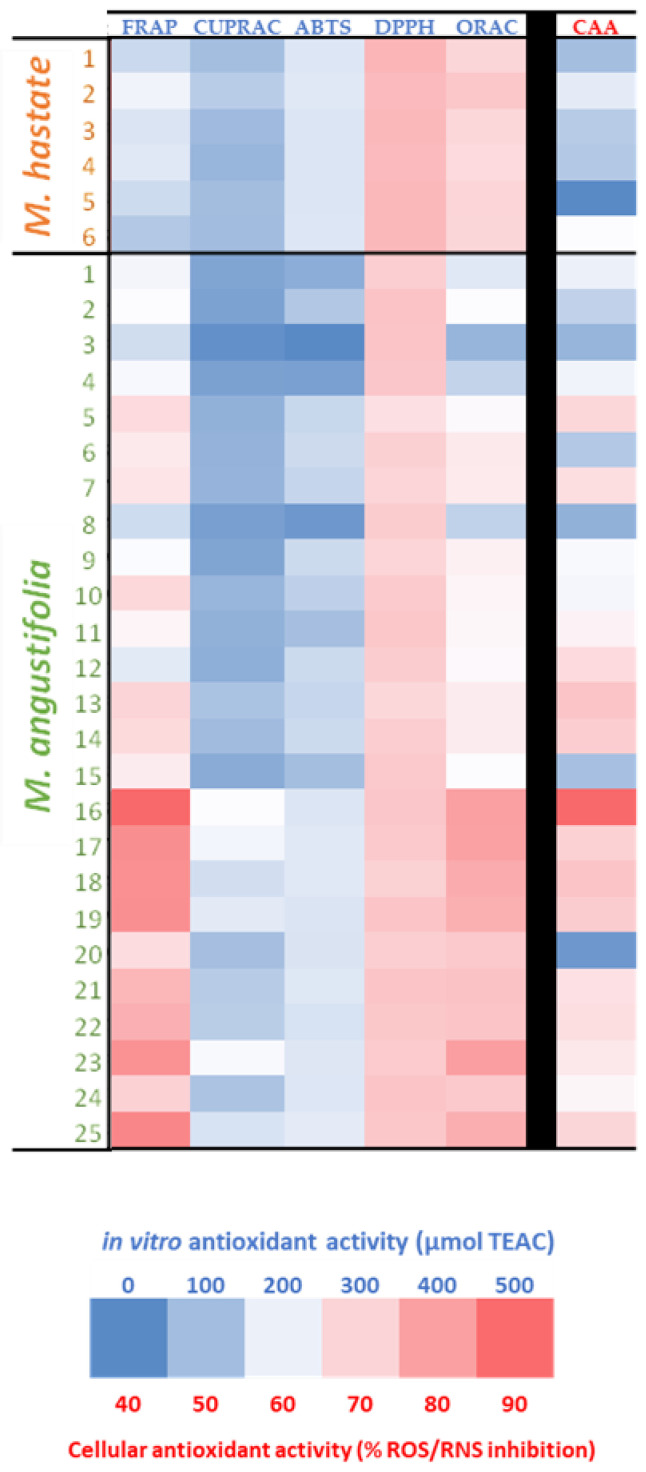
In vitro cell-free antioxidant (FRAP, CUPRAC, ABTS, DPPH and ORAC) and cellular antioxidant assay of extracts (CAA) from 25 different populations of *M. angustifolia* and 6 different populations of *M. hastata*. TEAC: TroloxC equivalent antioxidant capacity; ABTS: 2,2-azinobis (3-ethylbenzthiazoline-6-sulphonic acid); DPPH: 2,2-diphenyl-1-picrylhydrazyl; FRAP: ferric reducing antioxidant power; CUPRAC: cupric reducing antioxidant capacity; ORAC: oxygen radical absorbance capacity; CAA: cellular antioxidant assay. Means and standard deviations are provided in Table S2.

The antioxidant effect of the leaf extracts from various *M. angustifolia* and *M. hastata* populations were assessed for their antioxidant capacity using in vitro assays involving the two main antioxidant mechanisms such as the hydrogen atom transfer (HAT) mechanism assessed using ORAC assay and the single electron transfer (SET) mechanism assessed using CUPRAX and FRAP assays, whereas ABTS and DPPH assays allowed for both antioxidant mechanisms to be assessed [38,39,40]. The results expressed in µmol of Trolox-C equivalent antioxidant capacity (µmol TEAC) are summarized in Figure 7 and Table S2.

The antioxidant capacity differed substantially for the two species extracts. *M. hastata* showed high ORAC, DPPH and ABTS radical scavenging activities, suggesting the preponderance of a HAT antioxidant mechanism for these extracts. When compared to *M. hastata* extracts, *M. angustifolia* had a significantly higher FRAP antioxidant activity, in addition to a high DPPH radical scavenging activity, thus implying a greater contribution of the ET-based antioxidant mechanism. Furthermore, the HAT-based antioxidant capacity of *M. angustifolia* extracts is comparable to that of *M. hastata* extracts. As a result, by involving the two types of antioxidant mechanisms, *M. angustifolia* extracts appeared more attractive in terms of antioxidant capacity than *M. hastata* extracts.

The higher flavonoid contents of *M. angustifolia* extracts could be related to the largest contribution of the HAT mechanism. When it comes to quenching free radicals, flavonoids preferentially function through HAT-based reactions rather than ET-based reactions [25,41,42]. Other chemicals, notably phenolics, may contribute to this antioxidant effect in synergy via an ET-based mechanism [25,42]. Indeed, flavonoids are known to favor the HAT-based antioxidant mechanism over the ET-based antioxidant mechanism [25,41,42]. Here, other compounds, particularly phenolics, may also contribute in synergy with flavonoids via an ET-based mechanism [25,42]. For instance, Bai et al. [29] have reported the DPPH radical scavenging capacity of stigmasterol extracted from *M. hastata*.

The high antioxidant capacity of both *Monochoria* extracts was further confirmed in in vivo using the yeast model CAA assay, as the capacity to produce ROS and RNS in yeast cells subjected to UV-induced oxidative stress. A greater cellular antioxidant capacity was confirmed for *M. angustifolia* extracts as compared to *M. hastata* extracts.

### 3.4. Correlation Analysis

**Figure 8:**
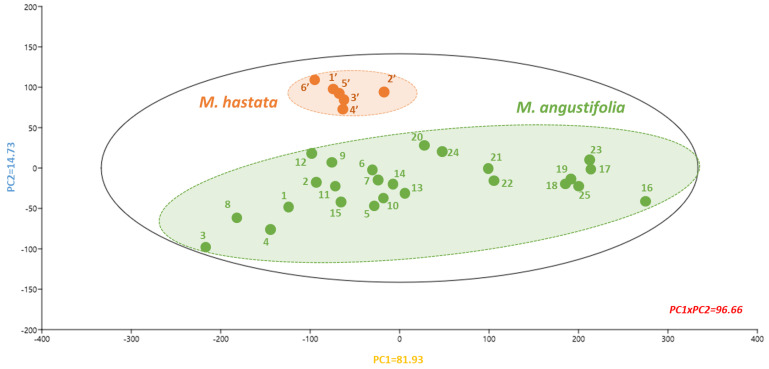
Principal component analysis linking the phytochemical profile as well as antioxidant capacity of extracts from different populations of the two *Monochoria* species. Variability of component 1 = 81.93% and component 2 = 14.73%. Each number represents the populations from *M. angustifolia* (in green) and *M. hastata* (in orange). The corresponding loading score plots for components 1 and 2 are presented in Figure S1.

The PCA or principal component analysis was used to identify relevant connections between the different natural populations based on their phytochemical composition and antioxidant activity (Figure 8).

Biplot representation accounted for 96.66% of the initial variability (Figure 8). The TFC and ET-based antioxidant FRAP and CUPRAC assays were the primary contributors to discriminate the component one axis, accounting for 81.93% of the initial variability (Figure S1). In contrast, the second axis accounted for just 14.73% of the initial variability with TPC and the mixed ET/HAT- and HAT-based antioxidant ABTS and ORAC assays (Figure S1). As a result of this PCA, two distinct clusters were discriminated from each other based on phytochemical composition as well as antioxidant capacity. Excitingly, these clusters separated *M. hastata* extracts from *M. angustifolia* extracts. The *M. hastata* extracts were grouped, whereas *M. angustifolia* extracts appeared substantially more diverse. 

**Figure 9:**
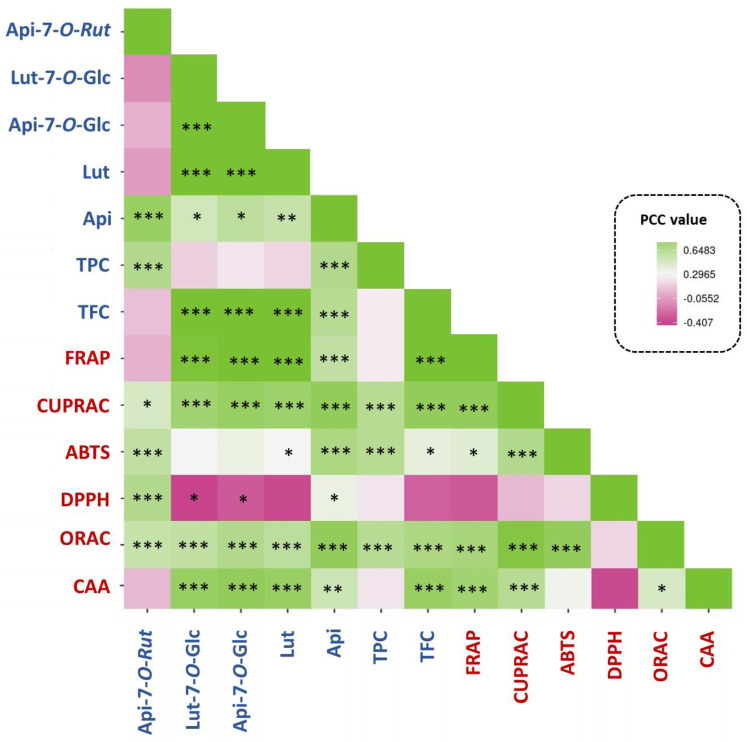
Pearson coefficient correlation (correlogram analysis) between the phytochemical profiles and antioxidant activity of extracts. Api-7-*O*-Rut: apigenin-7-*O*-rutinoside; Lut-7-*O*-Glc: luteolin-7-*O*-glucoside; Api-7-*O*-Glc: apigenin-7-*O*-glucoside (also known as apigetrin); Lut: luteolin; Api: apigenin; TPC: total phenolic content; TFC: total flavonoid content; ABTS: 2,2-azinobis (3-ethylbenzthiazoline-6-sulphonic acid); DPPH: 2,2-diphenyl-1-picrylhydrazyl; FRAP: ferric reducing antioxidant power; CUPRAC: cupric reducing antioxidant capacity; ORAC: oxygen radical absorbance capacity; CAA: cellular antioxidant assay. *** significant *p* < 0.001; ** significant *p* < 0.01; * significant *p* < 0.05; PCC values are indicated in Table S3.

Pearson correlation coefficients (PCC) were used to evaluate the linkage between phytochemicals and antioxidant capacity (Figure 9, Table S3).

The strength of various correlations between the phytochemicals (TPC, TFC and each flavonoid), and the various antioxidant assays was measured. The most remarkable were the highly significantly correlations between the various flavonoids and the ET-based antioxidant assays (FRAP and CUPRAC) and the cellular antioxidant assay, on the one hand, and the TPC and the HAT-based antioxidant assay, on the other. The correlations are consistent with the antioxidant mechanisms reported for these phytochemicals [25,42]. These correlations also highlighted that antioxidant capacity of the extract is typically the consequence of complex phytochemical combinations instead of the action of a single compound [43]. This study proved the great potential of *M. angustifolia* extracts as an alternative starting material for a variety of applications focused on their antioxidant flavonoids, as previously discussed in other plant species [9,10,13,27,37].

## 4. Conclusions

This current research on 25 populations of *M. angustifolia* collected from natural habitats in Thailand, exhibited high heterogeneity of phenolics/polyphenols accumulation. Likewise, this present study indicated that flavonoids are major phytochemicals of this plant, as well as demonstrating that *M. angustifolia* plant material is richer in flavonoids compared to the outgroup species from the same genus, *M. hastata*. The results showed that flavonoids might be employed to examine *Monochoria*’s evolutionary connections as well as for botanical authentication. Furthermore, the use of various in vitro antioxidant assays as well as a cellular antioxidant assay revealed that the antioxidant effect of *M. angustifolia* extracts is primarily facilitated by hydrogen atom transfer mechanisms, and antioxidant potential of the obtained extracts originated from complex phytochemical compounds. This study provides a new frontier of knowledge on the phytochemical diversity and antioxidant potential of *M. angustifolia* natural populations from all floristic regions in Thailand, harboring the highest species diversity in the *Monochoria* genus. This will certainly be an aid to phytopharmaceutical industries as this raw plant material has the potential to develop new bioactive products for the phytopharmaceutical sectors.

## Figures and Tables

**Table 1:** The collected 25 populations of the new species, *M. angustifolia* and outgroup (*M. hastata*).

Population No.	Population Names (The Collected Localities)	Floristic Regions	Scientific Name
1	Phichit	Northern (N)	*M. angustifolia*
2	Nakhon Sawan	Northern (N)	*M. angustifolia*
3	Khon Kaen	North-Eastern (NE)	*M. angustifolia*
4	Loei	North-Eastern (NE)	*M. angustifolia*
5	Chaiyaphum	Eastern (E)	*M. angustifolia*
6	Nakhon Ratchasima	Eastern (E)	*M. angustifolia*
7	Buri Ram	Eastern (E)	*M. angustifolia*
8	Ratchaburi	South-Western (SW)	*M. angustifolia*
9	Phetchaburi	South-Western (SW)	*M. angustifolia*
10	Prachuap Khiri Khan	South-Western (SW)	*M. angustifolia*
11	Saraburi	Central (C)	*M. angustifolia*
12	Suphan Buri	Central (C)	*M. angustifolia*
13	Nakhon Pathom	Central (C)	*M. angustifolia*
14	Rayong	South- Eastern (SE)	*M. angustifolia*
15	Prachin Buri	South- Eastern (SE)	*M. angustifolia*
16	Chachoengsao	South- Eastern (SE)	*M. angustifolia*
17	Sa Kaeo	South- Eastern (SE)	*M. angustifolia*
18	Chanthaburi	South- Eastern (SE)	*M. angustifolia*
19	Nakhon Si Thammarat	Peninsular (PEN)	*M. angustifolia*
20	Phatthalung	Peninsular (PEN)	*M. angustifolia*
21	Surat Thani	Peninsular (PEN)	*M. angustifolia*
22	Phangnga	Peninsular (PEN)	*M. angustifolia*
23	Trat	South- Eastern (SE)	*M. angustifolia*
24	Ranong	Peninsular (PEN)	*M. angustifolia*
25	Krabi	Peninsular (PEN)	*M. angustifolia*
Outgroup 1	Nakhon Sawan	Northern (N)	*M. hastata*
Outgroup 2	Loei	North-Eastern (NE)	*M. hastata*
Outgroup 3	Nakhon Ratchasima	Eastern (E)	*M. hastata*
Outgroup 4	Suphan Buri	Central (C)	*M. hastata*
Outgroup 5	Chachoengsao	South- Eastern (SE)	*M. hastata*
Outgroup 6	Ratchaburi	South-Western (SW)	*M. hastata*

**Note:** The population names come from the collected province/locality.

**Table 2:** Calibration function parameters for major flavonoids from *M. angustifolia* leaf extract using UV detection.

Flavonoid	Retention Time (t_R_)				Calibration Curve			LOD (µg/mL)	LOQ (µg/mL)
	Min	RSD (%)	R_s_	Sym Fact	Slope	Intercept	R^2^		
Api-7-Rut	19.90	0.76	3.33	1.07	2291.9	569.6	0.9996	0.08	0.25
Lut-7-Glc	29.19	0.20	3.63	1.01	2553.3	157.1	0.9994	0.02	0.06
Api-7-Glc	32.58	0.51	3.04	1.11	2468.8	642.5	0.9999	0.09	0.26
Lut	36.28	0.41	1.87	1.03	3670.2	285.1	0.9997	0.03	0.08
Api	40.56	0.24	1.67	0.98	3742.3	250.5	0.9992	0.02	0.07

Api: apigenin; Lut: luteolin; Api-7-Rut: apigenin-7-*O*-rutinoside; Lut-7-Glc: luteolin-7-*O*-glucoside; Api-7-Glc: apigenin-7-*O*-glucoside; RSD: relative standard deviation; R_s_: resolution value; Sym Fact: symmetry factor; R^2^: correlation coefficient; LOD: limit of detection; LOQ: limit of quantification.

**Table 3:** Quantification and validation parameters for the simultaneous analysis of the main flavonoids from *M. angustifolia* leaf ethanolic extract.

Flavonoid	Concentration	RSD (%)	HortRat	Accuracy		Intra-Day Precision		Inter-Day Precision	
	(mg/g DW)			Recovery (%)	RSD	%	RSD	%	RSD
Api-7-Rut	22.04 ± 0.02	0.09	0.03	100.32	1.34	99.15	3.88	99.85	0.67
Lut-7-Glc	18.91 ± 0.13	0.67	0.22	99.90	0.46	99.37	3.35	95.72	3.67
Api-7-Glc	36.84 ± 0.49	1.33	0.39	99.80	0.53	98.80	3.31	97.91	4.81
Lut	25.09 ± 0.15	0.58	0.18	100.11	0.41	99.64	1.43	98.77	4.97
Api	3.41 ± 0.20	5.93	2.47	98.40	3.10	97.99	4.31	96.98	4.37

Api: apigenin; Lut: luteolin; Api-7-Rut: apigenin-7-*O*-rutinoside; Lut-7-Glc: luteolin-7-*O*-glucoside; Api-7-Glc: apigenin-7-*O*-glucoside; RSD: relative standard deviation; HortRat: Horwitz ratio. Concentration values of *M. angustifolia* population #16.

## Data Availability

All the data supporting the findings of this study are included in this article and supplementary material.

## Supplementary Materials

The following supporting information can be downloaded at: https://www.mdpi.com/article/10.3390/antiox11050952/s1, Table S1. HPLC quantification (expressed in mg/100 g DW) of the main flavonoids in different populations of two *Monochoria* species (six populations of *M. hastata* and 25 populations of *M. angustifolia*) covering the entire floristic regions of Thailand; Table S2. *In vitro* cell-free antioxidant (FRAP, CUPRAC, ABTS, DPPH and ORAC) and cellular antioxidant (CAA) assays of extracts from 25 different populations of *M. angustifolia* and six different populations of *M. hastata*. Table S3. Pearson correlation coefficient linking phytochemicals and antioxidant activity of ethanolic extracts of different populations of two *Monochoria* species (six populations of *M. hastata* and 25 populations of *M. angustifolia*) covering the entire floristic regions of Thailand; Figure S1. Loading scores of the component 1 and component 2 of the PCA (presented in Figure 5) linking the phytochemical profile and antioxidant capacity of the extracts of *M. hastata* and *M. angustifolia* populations originating from various floristic regions of Thailand. 1. apigenin-7-O-rutinoside; 2. luteolin-7-O-glucoside; 3. apigenin-7-O-glucoside (also known as apigetrin); 4. Luteolin; 5. Apigenin; TPC: total phenolic content; TFC: total flavonoid content; ABTS: 2,2-azinobis (3-ethylbenzthiazoline-6-sulphonic acid); DPPH: 2,2-diphenyl-1-picrylhydrazyl; FRAP: ferric reducing antioxidant power; CUPRAC: cupric reducing antioxidant capacity; ORAC: oxygen radical absorbance capacity; CAA: cellular antioxidant assay.
