# Supplementary material for: Flavonoid Profiles and Antioxidant Potential of Monochoria angustifolia (G. X. Wang) Boonkerd & Tungmunnithum, a New Species from the Genus Monochoria C. Presl
Source: Antioxidants (Basel). 2022 May 12;11(5):952. doi: 10.3390/antiox11050952 (PMC9138080; doi:10.3390/antiox11050952)
Supplement: Supplementary file 1 [file antioxidants-11-00952-s001.zip › antioxidants-1689985-supplementary.pdf]

Supplementary Materials

Flavonoid Profiles and Antioxidant Potential of *Monochoria angustifolia* (G. X. Wang) Boonkerd & Tungmunnithum, a New Species from the Genus *Monochoria* C. Presl

Duangjai Tungmunnithum [1,2,3,*], Samantha Drouet [2], Laurine Garros [2], Jose Manuel Lorenzo [4,5], and Christophe Hano [2,3,*]

1 Department of Pharmaceutical Botany, Faculty of Pharmacy, Mahidol University, Bangkok 10400, Thailand; duangjai.tun@mahidol.ac.th (D.T.)
2 Laboratoire de Biologie des Ligneux et des Grandes Cultures, INRAE USC1328, Campus Eure et Loir, Orleans University, 28000 Chartres, France; duangjai.tun@mahidol.ac.th (D.T.) ; samantha.drouet@univ-orleans.fr (S.D.); laurine.garros@univ-orleans.fr (L.G.); hano@univ-orleans.fr (C.H.)
3 Le Studium Institue for Advanced Studies, 1 Rue Dupanloup, 45000 Orléans, France; duangjai.tun@mahidol.ac.th (D.T.) ; hano@univ-orleans.fr (C.H.)
4 Centro Tecnológico de la Carne de Galicia, Adva. Galicia n_ 4, Parque Tecnológico de Galicia, San Cibrao das Viñas, 32900 Ourense, Spain; jmlorenzo@ceteca.net (J.M.L.)
5 Área de Tecnología de los Alimentos, Facultad de Ciencias de Ourense, Universidad de Vigo, 32004 Ourense, Spain
* Correspondence: duangjai.tun@mahidol.ac.th (D.T.) ; hano@univ-orleans.fr (C.H.)

Table S1. HPLC quantification (expressed in mg/100g DW) of the main flavonoids in different populations of two *Monochoria* species (6 populations of *M. hastata* and 25 populations of *M. angustifolia*) covering the entire floristic regions from Thailand.

		(1)		(2)		(3)		(4)		(5)	
Species & population number		mean	SD	mean	SD	mean	SD	mean	SD	mean	SD
M. hastata	1	24.58	1.14	4.21	0.35	10.74	0.24	6.38	0.84	2.27	0.66
	2	28.15	2.90	4.94	0.13	12.29	0.06	7.28	0.21	2.60	0.17
	3	25.71	0.05	4.4	0.72	11.23	0.11	6.78	0.40	2.37	0.00
	4	25.96	0.75	4.32	2.99	11.34	1.55	6.63	0.54	2.40	0.04
	5	23.44	0.21	3.94	0.04	10.24	0.43	6.15	0.15	2.16	0.01
	6	19.78	1.12	3.43	0.17	9.63	0.26	5.77	0.92	1.83	0.06
M. angustifolia	1	8.40	0.20	7.23	0.12	14.03	1.60	9.52	0.64	1.30	0.02
	2	8.78	0.03	7.45	0.15	14.67	0.24	9.92	0.95	1.36	0.00
	3	6.94	0.12	5.91	0.36	11.59	0.94	7.86	0.38	1.07	0.01
	4	8.46	0.48	7.14	0.26	14.14	0.38	9.62	1.54	1.31	0.05
	5	10.71	0.73	9.28	1.38	17.89	0.58	12.14	2.35	1.65	0.07
	6	10.05	0.21	8.67	0.40	16.80	1.68	11.47	0.68	1.55	0.02
	7	10.41	0.13	8.91	0.90	17.41	1.03	11.81	0.41	1.61	0.01
	8	6.97	0.36	5.62	0.12	11.64	0.29	7.95	1.16	1.08	0.03
	9	8.60	1.11	7.34	0.21	14.38	2.62	9.76	0.78	1.33	0.01
	10	11.18	0.32	9.52	0.83	18.68	0.76	12.67	0.23	1.73	0.00
	11	9.27	0.05	7.67	0.43	15.50	0.13	10.56	0.39	1.43	0.04

	12	7.92	0.63	6.71	1.58	13.23	1.49	8.94	0.44	1.22	0.01
	13	11.44	0.28	9.98	0.33	19.12	0.67	12.98	0.20	1.77	0.10
	14	11.28	0.18	9.45	0.16	18.85	0.42	12.79	0.13	1.74	0.06
	15	9.91	0.66	8.56	0.48	16.57	1.56	11.23	0.46	1.53	0.15
	16	22.04	0.02	18.91	0.13	36.84	0.49	25.09	0.15	3.41	0.20
	17	16.33	0.76	14.11	0.29	27.30	1.81	18.53	0.54	2.52	0.62
	18	16.25	0.25	13.88	0.17	27.16	0.60	18.38	0.18	2.51	0.46
	19	16.61	0.31	14.29	0.72	27.76	0.75	18.74	0.22	2.57	0.10
	20	8.96	0.52	7.55	0.25	14.98	1.23	10.11	0.37	1.39	0.00
	21	13.60	0.47	11.54	1.15	22.73	1.11	15.23	0.33	2.10	0.02
	22	13.91	0.20	11.67	0.69	23.24	0.47	15.97	0.14	2.15	0.01
	23	15.10	2.03	12.97	0.32	25.24	0.74	17.33	0.62	2.33	0.16
	24	11.71	0.35	10.01	1.83	19.57	0.19	13.22	0.20	1.81	0.00
	25	14.74	0.09	12.55	5.08	24.64	4.39	16.72	7.63	2.28	0.11

1. apigenin-7-*O*-rutinoside, 2. luteolin-7-*O*-glucoside, 3. apigenin-7-*O*-glucoside (aka apigetrin), 4. luteolin, 5. apigenin.

Table S2. *In vitro* cell-free antioxidant (FRAP, CUPRAC, ABTS, DPPH and ORAC) and cellular antioxidant (CAA) assays of extracts from 25 different populations of *M. angustifolia* and 6 different populations of *M. hastata*.

Species - Population		FRAP (µmol TEAC)	CUPRAC (µmol TEAC)	ABTS (µmol TEAC)	DPPH (µmol TEAC)	ORAC (µmol TEAC)	CAA (% RO/NS inhibiion)
M. hastata	#1	168.82 ± 45.95 ^{fg}	126.98 ± 16.56 ^{cd}	189.42 ± 2.79 ^{bc}	359.52 ± 7.80 ^{a}	296.15 ± 20.66 ^{cd}	47.15 ± 1.56 ^{d}
	#2	209.72 ± 8.20 ^{f}	149.83 ± 6.07 ^{c}	192.34 ± 0.58 ^{bc}	347.14 ± 7.56 ^{a}	325.84 ± 4.67 ^{c}	51.29 ± 2.51 ^{cd}
	#3	187.00 ± 15.52 ^{fg}	122.11 ± 13.13 ^{d}	187.66 ± 2.92 ^{bc}	352.86 ±3.30 ^{a}	293.36 ± 14.72 ^{cd}	48.39 ± 2.56 ^{cd}
	#4	192.92 ± 11.34 ^{fg}	113.87 ± 12.46 ^{e}	187.95 ± 1.17 ^{c}	349.05 ±2.52 ^{a}	288.28 ± 13.32 ^{cd}	48.07 ± 3.96 ^{cd}
	#5	172.53 ± 15.21 ^{g}	126.61 ± 15.10 ^{cd}	187.95 ± 3.09 ^{bc}	352.86 ± 5.95 ^{a}	297.58 ± 13.27 ^{cd}	42.36 ± 5.12 ^{d}
	#6	143.75 ± 8.98 ^{g}	123.72 ± 10.24 ^{cd}	189.12 ± 2.03 ^{bc}	353.81 ± 1.90 ^{a}	295.67 ± 10.62 ^{cd}	52.82 ± 3.41 ^{cd}
M. angustifolia	#1	213.03 ± 16.31 ^{f}	89.19 ± 10.48 ^{ef}	100.82 ± 9.93 ^{fg}	310.95 ± 8.14 ^{de}	192.57 ± 17.59 ^{f}	51.81 ± 8.52 ^{bcd}
	#2	226.12 ± 29.34 ^{ef}	83.91 ± 22.43 ^{e}	141.75 ± 11.50 ^{ef}	332.86 ± 1.65 ^{b}	222.60 ± 31.57 ^{f}	48.96 ± 3.63 ^{cd}
	#3	175.70 ± 21.87 ^{g}	57.24 ± 11.74 ^{g}	46.43 ± 7.71 ^{fg}	330.95 ±6.67 ^{bc}	113.07 ± 18.13 ^{g}	46.28 ± 1.95 ^{cd}
	#4	217.30 ± 16.89 ^{f}	82.64 ± 16.47 ^{ef}	80.94 ± 5.60 ^{fg}	326.19 ±5.04 ^{bc}	160.29 ± 41.51 ^{fg}	52.06 ± 10.21 ^{bcd}
	#5	287.69 ± 30.07 ^{de}	107.51 ± 18.51 ^{e}	165.44 ± 26.03 ^{bc}	278.57 ± 7.56 ^{f}	228.97 ± 23.41 ^{f}	60.56 ± 7.32 ^{bc}
	#6	260.55 ± 14.66 ^{de}	110.65 ± 7.09 ^{e}	171.58 ± 9.96 ^{cd}	307.14 ± 14.29 ^{de}	260.81 ± 17.81 ^{de}	48.07 ± 5.62 ^{cd}
	#7	270.06 ± 18.95 ^{de}	112.45 ± 16.00 ^{ef}	162.81 ± 12.42 ^{cd}	297.62 ± 13.33 ^{ef}	258.30 ± 3.79 ^{ef}	59.45 ± 7.33 ^{bc}
	#8	173.22 ± 15.64 ^{g}	79.08 ± 10.78 ^{f}	68.65 ± 5.15 ^{fg}	315.71 ± 7.56 ^{d}	157.94 ± 15.44 ^{fg}	46.02 ± 9.91 ^{cd}
	#9	221.16 ± 19.06 ^{f}	89.38 ± 12.15 ^{ef}	170.99 ± 11.70 ^{bc}	298.57 ± 14.38 ^{e}	246.64 ± 10.25 ^{e}	52.59 ± 7.74 ^{bcd}
	#10	291.82 ± 33.68 ^{de}	114.32 ± 10.54 ^{ef}	155.20 ± 9.40 ^{bc}	318.57 ± 3.30 ^{d}	237.99 ± 29.02 ^{e}	52.34 ± 4.52 ^{bcd}
	#11	238.10 ± 14.61 ^{ef}	108.22 ± 8.97 ^{ef}	129.18 ± 38.89 ^{bcdefg}	325.24 ± 2.52 ^{c}	234.10 ± 49.00 ^{def}	55.14 ± 6.24 ^{bc}
	#12	194.99 ± 18.26 ^{fg}	103.65 ± 12.37 ^{ef}	170.99 ± 14.18 ^{bc}	314.76 ± 5.04^{de}	231.36 ± 13.24 ^{ef}	60.05 ± 7.89 ^{bc}
	#13	299.39 ± 22.41 ^{d}	134.55 ± 4.71 ^{d}	164.85 ± 15.04 ^{bcd}	294.76 ± 12.38 ^{f}	255.57 ± 10.47 ^{e}	64.34 ± 8.76 ^{bc}
	#14	289.20 ± 9.33 ^{d}	122.64 ± 6.48 ^{de}	170.70 ± 16.33 ^{bc}	312.86 ± 1.65 ^{ef}	255.39 ± 20.24 ^{de}	62.73 ± 3.65 ^{b}
	#15	256.28 ± 8.49 ^{e}	98.33 ± 6.30 ^{ef}	127.43 ± 29.07 ^{efg}	322.38 ± 3.81 ^{d}	224.80 ± 37.44 ^{ef}	47.36 ± 3.77 ^{cd}
	#16	503.66 ± 84.40 ^{ab}	225.45 ± 42.73 ^{ab}	187.95 ±1.78 ^{b}	326.19 ± 6.25 ^{cd}	400.79 ± 40.41 ^{a}	83.52 ± 2.14 ^{a}
	#17	433.83 ± 45.24 ^{abc}	212.45 ± 18.86 ^{ab}	192.34 ± 2.97 ^{ab}	321.43 ± 1.65 ^{d}	396.81 ± 17.00 ^{a}	61.89 ± 10.44 ^{bc}
	#18	430.66 ± 6.95 ^{ab}	177.09 ± 4.30 ^{ab}	192.63 ± 3.04 ^{ab}	304.29 ± 9.18 ^{ef}	377.00 ± 10.72 ^{a}	64.52 ± 8.12 ^{b}
	#19	431.21 ± 22.31 ^{ab}	196.23 ± 23.88 ^{ab}	185.91 ± 5.87 ^{bc}	329.05 ± 6.87 ^{bcd}	370.75 ± 19.23 ^{ab}	62.89 ± 6.01 ^{bc}
	#20	284.24 ± 42.75 ^{def}	129.30 ± 17.44 ^{cd}	185.03 ± 2.88 ^{b}	310.00 ± 1.65 ^{e}	322.29 ± 11.81 ^{c}	43.69 ± 6.51 ^{cd}
	#21	357.93 ± 31.24 ^{cd}	148.14 ± 11.38 ^{cd}	190.88 ± 2.53 ^{bc}	328.10 ± 5.30 ^{bcd}	333.98 ± 13.06 ^{bc}	58.75 ± 9.32 ^{bc}
	#22	370.06 ± 30.30 ^{bc}	150.20 ± 14.76 ^{cd}	182.98 ± 5.29 ^{b}	323.33 ± 5.79 ^{cd}	330.08 ± 14.83 ^{bc}	59.23 ± 9.18 ^{bc}
	#23	429.01 ± 25.39 ^{ab}	218.29 ± 13.71 ^{a}	190.00 ± 1.83 ^{bc}	317.62 ± 10.73 ^{cde}	402.57 ± 7.15 ^{a}	57.21 ± 6.37 ^{bc}
	#24	305.87 ± 26.63 ^{d}	135.63 ± 24.21 ^{cd}	189.12 ±2.21 ^{bc}	329.05 ± 5.04 ^{cd}	320.38 ± 24.26 ^{c}	54.36 ± 8.31 ^{bcd}
	#25	447.74 ± 38.89 ^{ab}	183.31 ± 10.51 ^{b}	196.73 ±0.77 ^{a}	325.24 ± 4.15 ^{cd}	373.19 ± 8.46 ^{ab}	60.86 ± 8.23 ^{bc}

TEAC: TroloxC equivalent antioxidant capacity; ABTS: 2,2-azinobis (3-ethylbenzthiazoline-6-sulphonic acid; DPPH: 2,2-diphenyl-1-picrylhydrazyl; FRAP: ferric reducing antioxidant power; CUPRAC: cupric reducing antioxidant capacity; ORAC: oxygen radical absorbance capacity; CAA: cellular antioxidant assay. Different superscript letters indicate significant differences at $p < 0.05$.

Table S3. Pearson correlation coefficient linking phytochemicals and antioxidant activity of ethanolic extracts of different populations of two *Monochoria* species (6 populations of *M. hastata* and 25 populations of *M. angustifolia*) covering the entire floristic regions from Thailand.

	(1)	(2)	(3)	(4)	(5)	TPC	TFC	FRAP	CUPRAC	ABTS	DPPH	ORAC
**(1)**												
**(2)**	-0.101											
**(3)**	0.0176	0.992***										
**(4)**	-0.0502	0.998***	0.998***									
**(5)**	0.806 ***	0.507*	0.606*	0.551**								
**TPC**	0.686 ***	0.141	0.218	0.174***	0.673							
**TFC**	0.0812	0.983***	0.998***	0.991***	0.656	0.259						
**FRAP**	0.030	0.968***	0.977***	0.974***	0.603	0.262	0.976					
**CUPRAC**	0.467*	0.750	0.811***	0.780***	0.850***	0.656***	0.836	0.844				
**ABTS**	0.601***	0.290	0.364*	0.320***	0.691***	0.642*	0.398*	0.425***	0.681			
**DPPH**	0.690***	-0.407*	-0.317*	-0.364	0.360*	0.228	-0.275	-0.317	0.0631	0.157		
**ORAC**	0.564***	0.600***	0.673***	0.633***	0.846***	0.660***	0.704***	0.731***	0.938***	0.855***	0.162	
**CAA**	0,052	0,820***	0,833***	0,828***	0,533**	0,376	0,233***	0,830***	0,756***	0,648	0,331	-0,352*

*** significant $p < 0.001$; ** significant $p < 0.01$; * significant $p < 0.05$; (1) apigenin-7-*O*-rutinoside ; (2) luteolin-7-*O*-glucoside ; (3) apigenin-7-*O*-glucoside (aka apigetrin) ; (4) luteolin ; (5) apigenin ; TPC: total phenolic content; TFC: total flavonoid content; FRAP: *in vitro* antioxidant FRAP assay; CUPRAC: *in vitro* antioxidant CUPRAC assay; ABTS: *in vitro* antioxidant ABTS assay; DPPH: *in vitro* antioxidant ABTS assay; ORAC: *in vitro* antioxidant ORAC assay; CAA: cellular antioxidant assay.

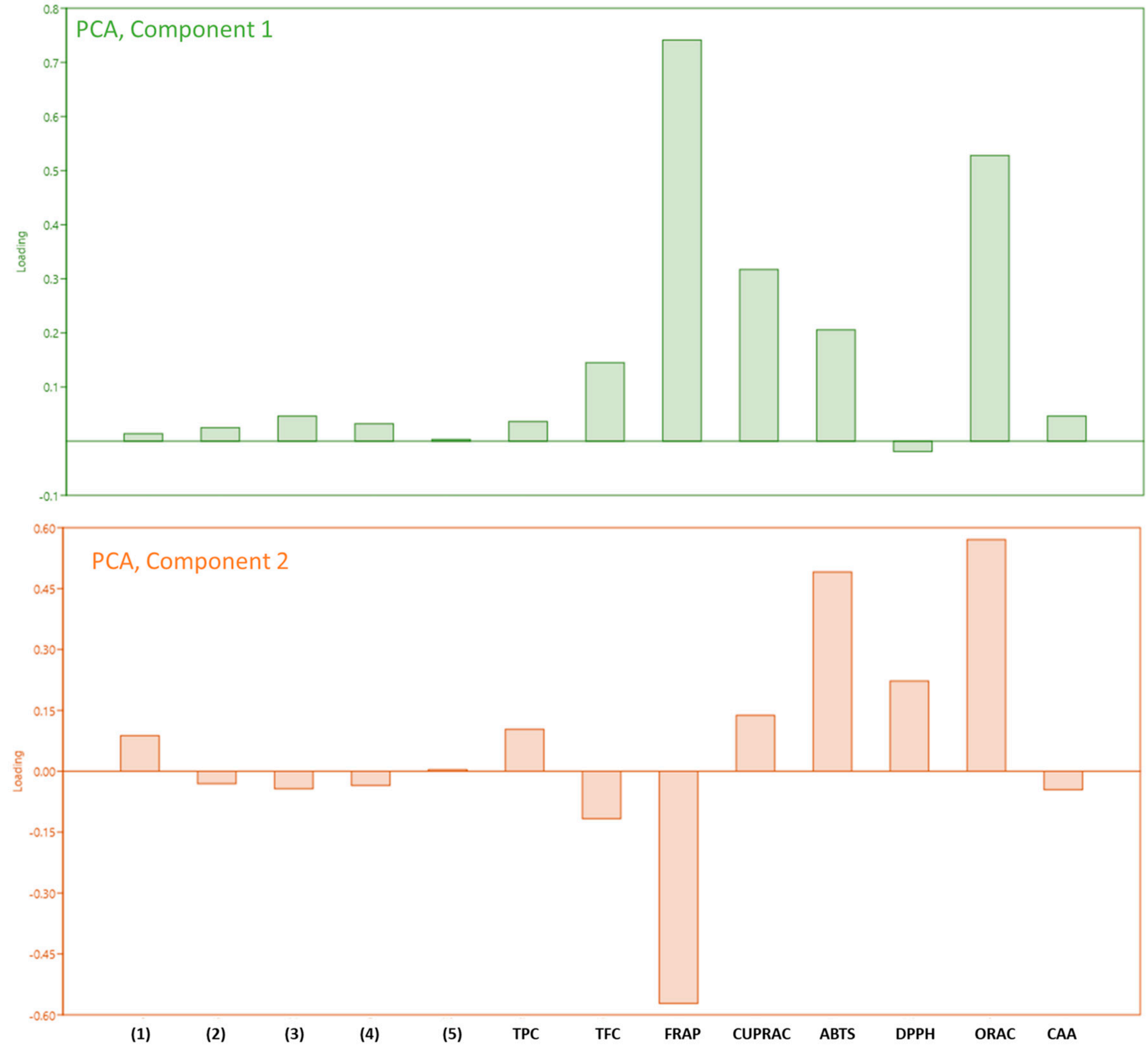

Figure S1. Loading scores of the component 1 and component 2 of the PCA (presented in Figure 5) linking the phytochemical profile and antioxidant capacity of the extracts of *M. hastata* and *M. angustifolia* populations originating from various floristic regions from Thailand. 1. apigenin-7-*O*-rutinoside; 2. luteolin-7-*O*-glucoside; 3. apigenin-7-*O*-glucoside (aka apigetrin); 4. Luteolin; 5. Apigenin; TPC: total phenolic content; TFC: total flavonoid content; ABTS: 2,2-azinobis (3-ethylbenzthiazoline-6-sulphonic acid; DPPH: 2,2-diphenyl-1-picrylhydrazyl; FRAP: ferric reducing antioxidant power; CUPRAC: cupric reducing antioxidant capacity; ORAC: oxygen radical absorbance capacity; CAA: cellular antioxidant assay.
